# Corrigendum: The disease associated Tau35 fragment has an increased propensity to aggregate compared to full-length tau

**DOI:** 10.3389/fmolb.2023.1276677

**Published:** 2023-09-15

**Authors:** Chen Lyu, Stefano Da Vela, Youssra Al-Hilaly, Karen E. Marshall, Richard Thorogate, Dmitri Svergun, Louise C. Serpell, Annalisa Pastore, Diane P. Hanger

**Affiliations:** ^1^ Department of Basic and Clinical Neuroscience, King’s College London, London, United Kingdom; ^2^ European Molecular Biology Laboratory, Hamburg Site, Hamburg, Germany; ^3^ Sussex Neuroscience, School of Life Sciences, University of Sussex, Brighton, United Kingdom; ^4^ London Centre for Nanotechnology, University College London, London, United Kingdom

**Keywords:** biophysical studies, hybrid methods, tauopathy, tau truncation, small angle x-ray scattering, intrinsically disordered proteins, neurodegeneration, dementia

In the published article, there was an error in the caption for Figure 1. The column used for size-exclusion chromatography was incorrectly stated. The corrected caption appears below:

**FIGURE 1:**
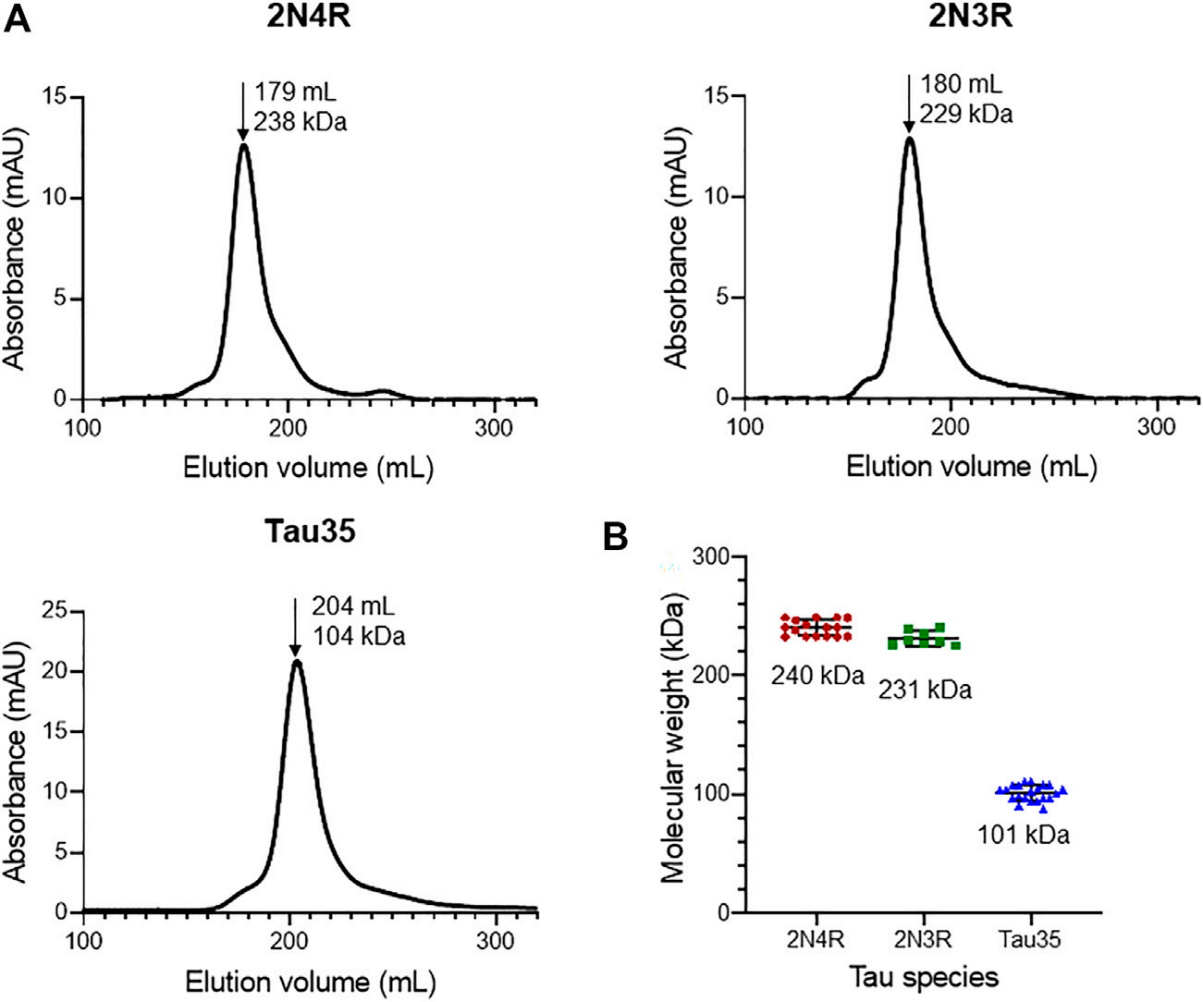
Size-exclusion chromatography of recombinant tau species. Gel filtration (HiLoad 26/600 Superdex 200 pg column) of recombinant 2N4R tau, 2N3R tau, and Tau35. **(A)** Representative elution profiles showing apparent molecular weights calculated from the elution volumes (arrows) of each tau species in comparison to a standard calibration curve. **(B)** Scatter plots showing the range of apparent molecular weights determined for each tau species. Bars indicate mean ± SD, *n* ≥ 8.

In the published article, there was an error. The column used for size-exclusion chromatography was incorrectly stated. A correction has been made to **Results**, *Characterizing the Tau35 Conformation Ensemble by Small-Angle X-Ray Scattering*, paragraph 1. This sentence previously stated:

“The apparent molecular weights of the tau species in phosphate buffered saline (PBS) were estimated from their elution volumes from a Superdex 200 10/300 column (Figure 1A) in comparison to globular protein standards.”

The corrected sentence appears below:

“The apparent molecular weights of the tau species in phosphate buffered saline (PBS) were estimated from their elution volumes from a HiLoad 26/600 Superdex 200 pg column (Figure 1A) in comparison to globular protein standards.”

A second correction has been made to **Experimental Procedures**, *Size-Exclusion Chromatography Measurements*. This sentence previously stated:

“Analytical SEC was carried out using a Superdex 200 10/300 GL column (Cytiva), pre-calibrated using a gel filtration marker kit (WMGF200) based on globular proteins.”

The corrected sentence appears below:

“Analytical SEC was carried out using a HiLoad 26/600 Superdex 200 pg column (Cytiva), pre-calibrated using a gel filtration marker kit (WMGF200) based on globular proteins.”

The authors apologize for this error and state that this does not change the scientific conclusions of the article in any way. The original article has been updated.

