# Supplementary material for: The Disease Associated Tau35 Fragment has an Increased Propensity to Aggregate Compared to Full-Length Tau
Source: Front Mol Biosci. 2021 Oct 28;8:779240. doi: 10.3389/fmolb.2021.779240 (PMC8581542; doi:10.3389/fmolb.2021.779240)
Supplement: Supplementary file 1 [file DataSheet1.docx]

**Supplementary Materials**

**Figure S1. The Guinier plots of 2N4R tau, 2N3R tau and Tau35.**


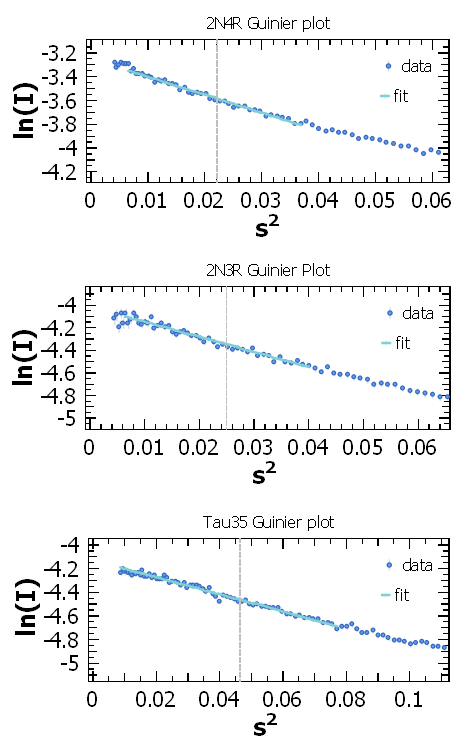


**Figure S2. Pair distance distribution functions [P(r)] for 2N4R tau, 2N3R tau and Tau35.** The data suggest the presence of elongated conformations for all three proteins.


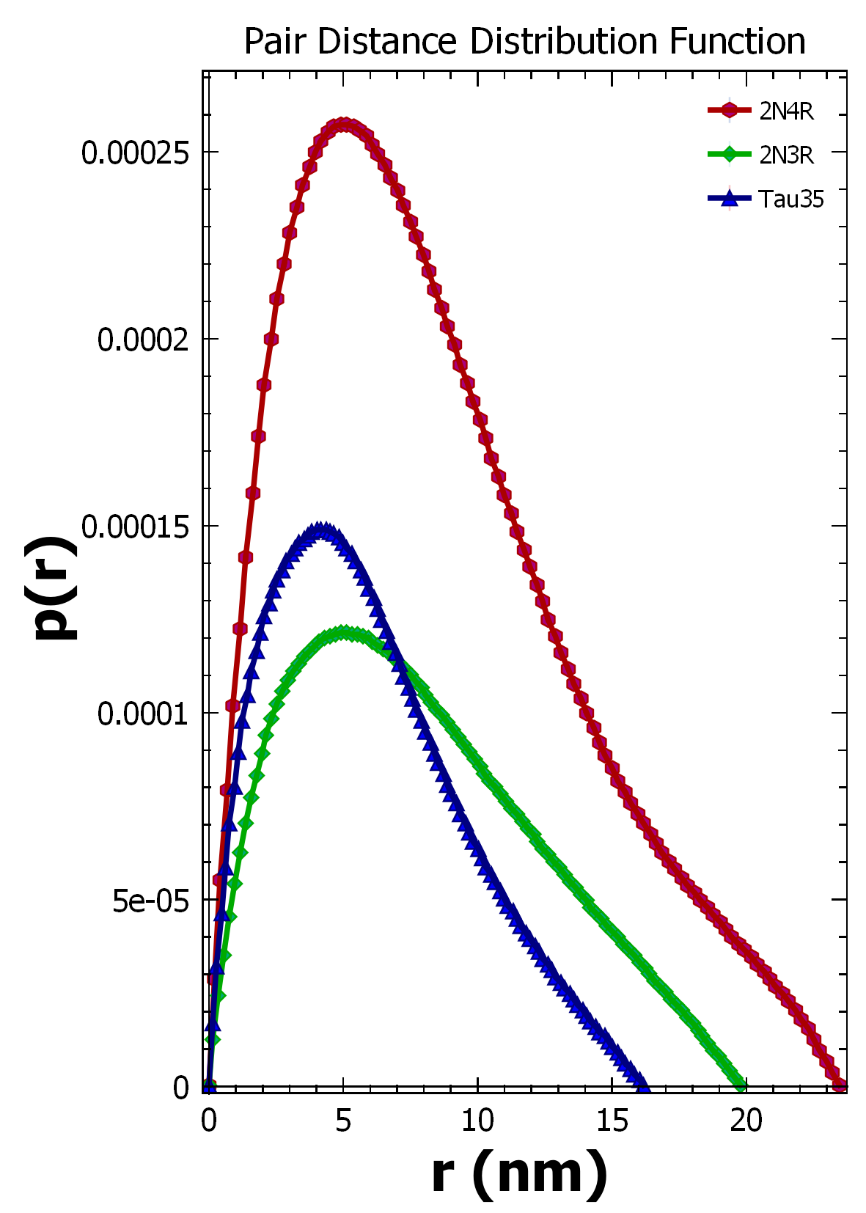


**Figure S3. Monodisperse Gaussian coil model for 2N4R tau, 2N3R tau and Tau35.** The data display the fitting of 2N3R tau, 2N4R tau and Tau35 with the monodisperse Gaussian coil model. Rg, Radius of gyration.


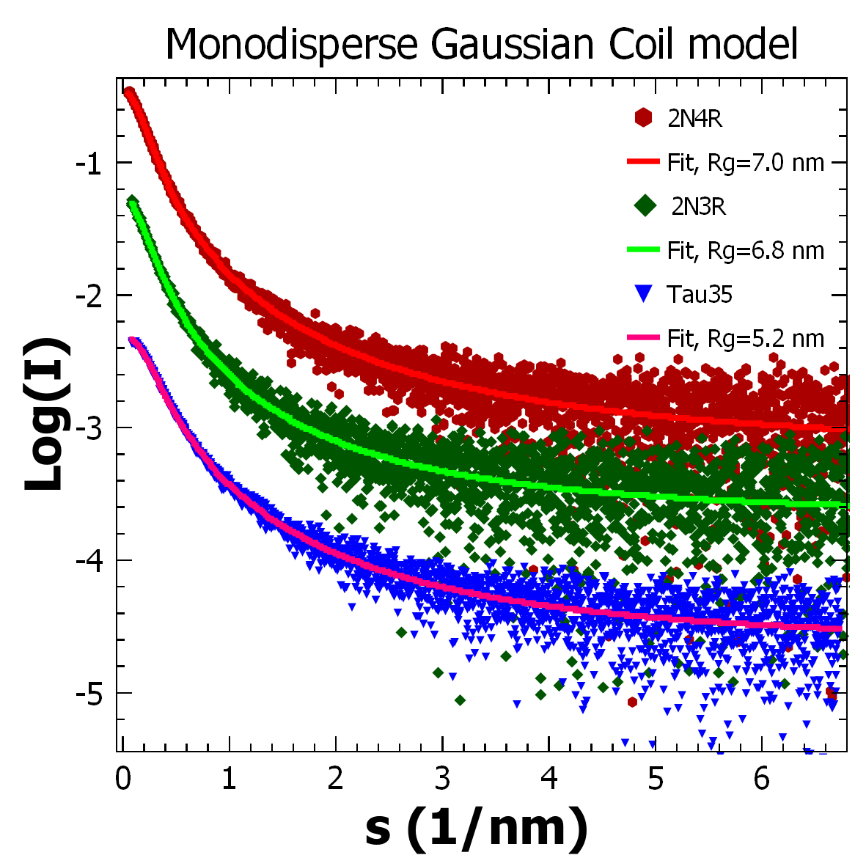


**Figure S4. Volume of correlation scaling of 2N4R tau, 2N3R tau and Tau35.** The proteins do not cluster with globular proteins in a double logarithmic plot of the volume of correlation against the number of amino acids. The ensemble-averaged radii of gyration can be obtained directly from the data, using the Guinier approximation.


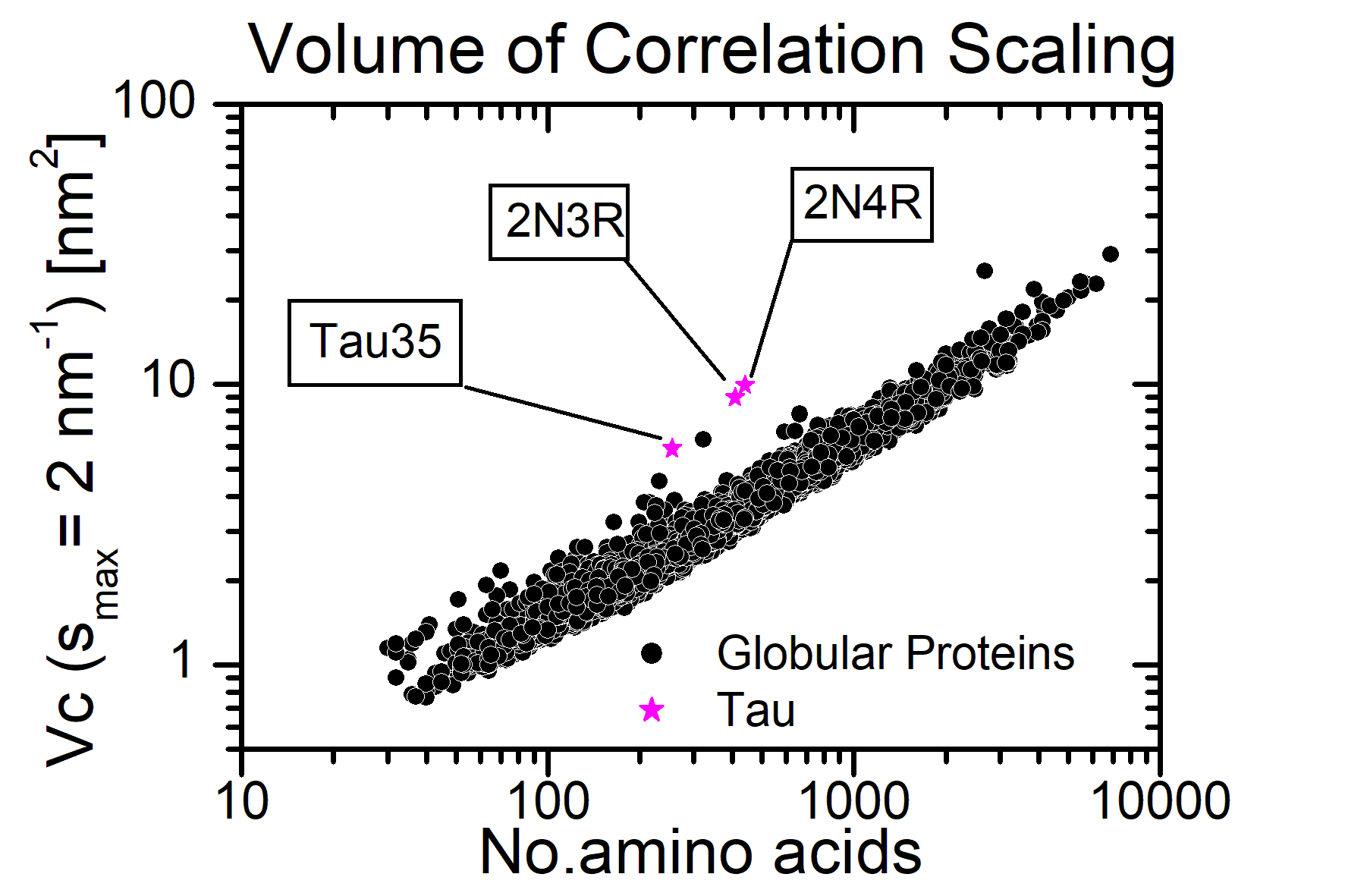


**Figure S5. CD spectra of recombinant 2N4R tau, 2N3R tau and Tau35 in PBS without aggregation induction.** The spectra exhibit features typical of random coil structures.
